# Reforming the veteran: propaganda and agency in the First World War Reconstruction hospitals

**DOI:** 10.5195/jmla.2019.743

**Published:** 2019-10-01

**Authors:** Aaron Jackson

**Affiliations:** Doctoral (PhD) Candidate, Department of Anthropology, History & Social Medicine, University of California, San Francisco, San Francisco, CA, aaron.jackson@ucsf.edu

## Abstract

The United States’ entry into the First World War prompted progressives to reform veterans’ entitlements in the hopes of creating a system insulated from corruption and capable of rehabilitating disabled veterans into productive members of society. The replacement of pensions with medical care for wounded and disabled soldiers through the Reconstruction Hospital System was originally intended as a temporary measure but resulted in establishing the foundations of the modern veterans’ health care system. Yet, these reforms would not have been possible without the support from the community of war veterans to which these reforms applied. By examining the communal values expressed in publications produced by and for soldiers, this paper explores the ways in which the Great War’s veteran community expressed agency in the process of reforming the US veteran entitlements.

## INTRODUCTION

In December 1918, President Woodrow Wilson traveled to France aboard the USS *George Washington* to take part in peace conferences to end the Great War. It was a momentous occasion, marking the first overseas trip by a sitting American president. It was also a hopeful occasion, for Wilson had a plan to establish world peace based on progressive logic. His “Fourteen Points” represented continued faith in human progress: that the world, indeed that humanity itself, could be improved through the application of scientific principles supported by strong moral foundations.

To contemporary observers, progressive reforms worked. They reduced corruption, inspired competition, improved workplace safety, boosted productivity, and supported ideals about the family—all with the support of emerging expertise in the sciences. In the war itself, scientific expertise and innovation led to enormous success. Propaganda campaigns successfully sold the war to a skeptical public, economists advised the government in efficient resource management, and medical experts’ innovations meant more soldiers survived the traumas of warfare than previous generations—one remarkable success that had the potential to cause different problems.

Fearing the potential costs associated with a generation of wounded veterans who would be dependent on the government, officials set out to apply progressive principles in reforming veterans’ entitlements and rehabilitating disabled soldiers. It was a grand experiment that resulted in the formation of the structures that would become the modern veterans’ health care system. But for that structure to stand, it needed a strong foundation, which was formed in the communities of veterans of an often-overlooked phase of the First World War.

The United States’ entry into the First World War prompted progressives to reform veterans’ entitlements in the hopes of creating a system that was insulated from corruption; was capable of turning disabled veterans into productive, independent members of society; and replaced pensions with medical care for wounded and disabled soldiers through the Reconstruction Hospital System. But these reforms would not take hold without support from the community of war veterans to which these reforms applied. By examining the communal values expressed in publications produced by and for soldiers, this paper explores the ways in which the Great War’s veteran community expressed agency in the process of reforming the US veteran.

## A MOMENTOUS OCCASION: THE ARMISTICE AND THE END OF THE GREAT WAR

As President Wilson made his way to the Paris Peace Conference, the crew of the *George Washington* marked what was, for them, an even more momentous occasion than transporting the commander in chief to Europe. After a full year of ferrying more than 50,000 troops and many thousand tons of cargo from the United States to support the war effort in France, on December 16, 1918, the “President’s Ship” was, for the first time, taking Americans home again. Chaplain P. F. Bloomhardt commented on the occasion in the ship’s newspaper:

At last THE DAY has arrived. Ten times have troops disembarking from this ship heard the familiar farewell, “Some day we will be bringing you back and that will be some joy ride with lights on and smoking permitted on deck—a regular time, homeward bound.” To this DAY, the men on this ship have been looking forward for almost exactly one year and now it has come. With it, is our welcome. [1]

The *George Washington* already had a long history by that point. Originally a German luxury liner equipped with the latest amenities, the ship dropped anchor in the then-neutral harbor of New York at the start of the war in 1914. After the United States’ declaration of war on Germany in 1917, the Americans seized her and converted her into a troop carrier capable of carrying over 6,000 passengers and crew. The Navy even converted a portion of the ship’s hold into a hospital capable of caring for as many as a thousand wounded soldiers at a time [2]. And while the *George Washington* boasted a wireless telegraph and printing press to provide passengers with the latest news and entertainment, their use was so strictly censored during the war that the crew were not allowed to mention even the name of their ship in print for fear that such information would fall into enemy hands. But with the declaration of the armistice, the military lifted most censorship restrictions, and the crew began printing their own newspaper to entertain themselves and the passengers they were carrying home. In keeping with the theme of the ship’s namesake, they called it *The Hatchet*.

The lead headline in *The Hatchet*’s first edition on December 16, 1918, proudly proclaimed, “Three Thousand Troops and Nine Hundred Wounded Home by Christmas!” [3]. The distinction between the two groups in that headline is telling. Technically, the wounded were also troops, meaning there was no need to differentiate between the two groups.

But in their effort to boast about their ship and play to the patriotic sacrifices of the wounded, *The Hatchet*’s editorial staff perhaps inadvertently revealed that the two groups were separated by more than their location in the ships’ holds. The “troops” were bound for ticker tape parades in New York City, holiday leave for the New Year, speedy discharges, and train tickets home. The “wounded” and those personnel responsible for their care, however, were merely beginning a new episode of the war—one that would continue in the wards and barracks of the US Army general hospital system, and one that the Army Medical Corps would later characterize as the longest, hardest battle of the First World War [4]. This new phase, the Reconstruction phase, would fundamentally reshape US veterans—physically, politically, and socially—and those veterans, in turn, would cement the foundations of the modern veterans’ health care system.

Historians have written a great deal about the First World War, but the vast majority of the historiography focuses on the great personalities: the heads of state and policy makers who set the agenda, the generals who gave the orders and developed strategies, and the surgeons and scientists whose wartime innovations changed the war. This remains true despite a wealth of new historiography inspired by the war’s centenary.

The contributions and experiences of the common soldiers, however, have remained largely obscured outside of literature. Memoirs and reflective works such as Robert Graves’s *Good-Bye to All That* (1929) or Helen Zenna Smith’s *Not So Quiet…* (1930) provide vivid accounts, but these works were often written years after the guns fell silent, introducing potential problems of memory and postscript editorializing in addition to fictional insertions that make it difficult to tell the difference between fact and embellishment. Personal correspondence and journals provide excellent contemporary insight but being limited to an individual and perhaps their immediate circle, they can be difficult to place in larger, collective contexts. Thankfully, there are sources that illuminate Great War veterans’ contemporary communal experiences in context: what have come to be colloquially called trench journals.

Trench journals consist of a vast array of publications by the soldiers of every nation involved in the war, often without official sponsorship. Long neglected as historical sources, these newspapers and magazines have only recently garnered serious scholarly attention. J. G. Fuller pioneered trench journal scholarship in 1990 with an exploration of newspapers produced by the British and Dominion Armies [5]. In 1995, Stéphane Audoin-Rouzeau studied the effects of national sentiment in France as reflected in French trench journals [6]. Robert L. Nelson provided the first systematic study in English of German soldier newspapers in 2011 [7]. And Graham Seal’s excellent work on the newspapers of the Australian and New Zealand Army Corps was published in 2013 [8].

Yet, scholarship on American journals remains relatively scant, likely due to the fact that American units rarely published such newspapers during the war itself. American publications during the war tended to be of the official variety, such as the *Stars and Stripes* or the surgeon general’s magazine, *Carry On*. It was not until after the war ended and officials lifted censorship restrictions that American units began printing newspapers that provided the type of insight found in the British, French, and German trench journals. Published by and for members of various military communities, including Reconstruction hospitals, these sources provide unique windows into the contemporary views and values of the groups that produced and consumed them. As such, they represent a unique means of examining the cultures of war and military medicine.

The terms “trench journal” and “unit magazine” are not necessarily interchangeable. Unit magazines were most often produced through official channels, published professionally, and sanctioned by the chain of command. Conversely, trench journals were almost exclusively published without official support on an ad hoc basis as conditions allowed on the front lines and often as response to troops’ perceived failures in their military and political leadership, which they blamed for the war itself, and in the popular media, which they viewed as little more than jingoistic propaganda. American Reconstruction hospital newspapers tended toward a mixture of these two types. Often granted official sanction and published in a professional manner, these newspapers nevertheless provided outlets for wounded soldiers and members of the Medical Corps to express agency in a system whose mechanisms were often beyond their control.

## TRENCH JOURNALISM: BUILDING COMMUNITIES IN THE TRENCHES

In Europe, trench warfare was a necessary precondition for trench journals to be produced, as soldiers on the move could not lug around the heavy equipment required to produce even rudimentary newspapers. But for entrenched combatants with access to abandoned civilian equipment, publication was entirely possible. After October 1914, when belligerent armies began digging trenchworks that stretched across Europe from the Alps to the North Sea and under varying circumstances in each case, newspapers began to appear in Allied units. In his first editorial of *The Wipers Times*—one of the longest-running and best-known trench publications—Captain F. J. “Fred” Roberts of the British Expeditionary Force explained how his journal came about:

Having managed to pick up a printing outfit (slightly soiled) at a reasonable price, we have decided to produce a paper. There is much that we would like to say in it, but the shadow of censorship enveloping us causes us to refer to the war, which we hear is taking place in Europe, in a cautious manner. [9]

Captain Roberts’s tongue-in-cheek humor was often a clever way of bypassing the censor. Potentially serious information, such as revealing the unit was stationed at a place that had a printing press in Ypres, Belgium, which the British called “Wipers,” could be written off as mere amusement. Comedy, however, was not always a shield against the censor.

Like every other form of communication in the war, military commands subjected trench journals to censorship, resulting in the suppression of an unknown number of these publications, which is one of the reasons why modern scholars have such a difficult time identifying the precise distribution and impact of these works. But as the war ground on and as commanders grew increasingly concerned with maintaining morale, Allied high command decided to allow these journals because they appeared to boost soldiers’ spirits. General Joseph Joffre, commander-in-chief of Allied Forces in 1916, issued a memorandum in March of that year directing military censors to allow trench journal publication, provided that “their management is closely supervised” [10]. Thereafter, editors of prospective journals often worked closely with the censors to avoid unwanted complications, and good officers learned to use the newspapers to keep a pulse on their men’s concerns and morale.

Soldiers in the trenches subscribed to trench newspapers because they provided a rare commodity in the war: entertainment. A profusion of stresses marked life on the front lines. The troops had to deal with snipers, artillery, gas, machine guns, and a cacophony of other hazards. It was difficult to rest in flooded trenches, hard to stay warm when they could not light a campfire for fear of attracting gunfire, and impossible to relax as pests—most often rats and lice—insisted on interrupting any moment of repose.

These physical stresses added up, and they were compounded by the true scourge of military life: the ever-present boredom. Long days of repetitive routine with nothing to look at but muddy walls and a strip of sky overhead left soldiers seeking anything they could find for entertainment. Trench newspapers provided fresh fodder, supplying soldiers with momentary escapes from their drudgery with a reminder that their burdens and tortures were not borne alone: others were going through the same things and somehow finding sardonic humor in it all, as demonstrated by an “advertisement” from *The Wipers Times*:

Nightly,

THE GREAT SPECTACULAR PICTURE

**INFERNO**

50,000 Artistes have been engaged to

produce this colossal work, at enormous

expense. Music and effects of this great

picture by the International Orchestra [11]

Other advertisements promoted all-expense-paid circular tours “embracing all the health resorts of lovely Belgium,” entreating interested parties to seek tickets from “R.E. Cruiting & Co., London. Agents everywhere,” [12] or medical advice columns asking readers if they were victims of optimism:

Do you suffer from cheerfulness? Do you sometimes think that the war will end within the next twelve months? If your answer is “yes”…then you are in the clutches of that dread disease. We can cure you. Two days spent at our establishment will effectively eradicate all traces of it from your system. [13]

Describing life on the front lines as if it were a less-lethal modern novelty like a motion picture show or the trenches were part of a tour of Belgium’s health resorts, or framing optimism as a dreaded disease exemplified the gallows humor often found in trench journals. Humor disarmed more than the censor, after all. It undermined the sense of isolation and reminded soldiers that their experiences were shared among the brotherhood of frontline soldiers. And the fact that someone could find a joke to tell in that common experience of suffering built bonds in the soldier-families that formed in the trenches.

Trench journals provided another crucial form of entertainment by circulating rumors and gossip. Correspondence home took weeks and was often intercepted by the censors, troops widely distrusted and mocked mainstream newspapers as jingoists, and general headquarters stayed generally quiet about the goings-on elsewhere along the front or at home. Servicemen, thus, sought what they perceived to be more reliable sources of information: their fellow soldiers. How was the war going? Who was getting leave and when? What would happen when the war ended? With the help of readers’ contributions, trench journals had answers [14]. It did not matter that these contributions were as dubious as any other rumor exchanged in the trenches; information in the journals was at least generated by, for, and about frontline soldiers themselves, which was vastly preferable to what many considered the rubbish coming from the brass, press, and propagandists.

Soldier-produced publications also provided a much-needed outlet for the age-old soldierly tradition of complaining. They grumbled about the daily discomforts—primarily the mud, lice, officers, and food, usually in that order—but they often reserved their most severe complaints for those who failed to do their duty as the soldiers saw it. Most of the troops were conscripts who, given the chance, would have avoided the war; yet they took resolute pride in the fact that they served and in the bonds of brotherhood that they formed. These bonds were unusually strong, born out of mutual hardship and shared suffering, as the following poignant piece suggests:

Having labored together, marched together, suffered at the same places…having bent their heads under the same rain, having suffered the blast from the same heavy shells, they developed a deep friendship for each other…Mutual friendship sustains them through their long and harsh labour; like oxen tied to the same yoke, each feels the support of the other and suffers less because they know they are suffering together. [15]

Thus, trench journals served as communal interpretations of acceptable and unacceptable behavior. Comrades became heroes of varying sorts. The man who frustrated the brass at every turn with his wit was someone to be admired. The bunkmate who died in the shelling the other day was someone worthy of somber remembrance. The quartermaster was the gatekeeper of all troglodytic pleasures. Even the enemy could be more relatable than one’s own high command: sure, he might be a “Bosche,” but he was stuck in the trenches because some brass hat or some politician told him he had to be there, just like everyone else [16]. But the jingoists and shirkers back home were universally despised and often described with a palpable vitriol.

These publications were the result of long processes and hard lessons learned in the barbaric conditions of the Western Front. When the United States joined the war in 1917, American leadership had the advantage of drawing on the Allies’ experiences and examples in determining the development and direction of American publications and propaganda.

## AMERICAN PROPAGANDA: PROMOTING FAITH IN THE CAUSE

General John J. Pershing, commander-in-chief of the American Expeditionary Forces (AEF) in France, desired a publication that would boost morale as the trench journals had done for the Allies and one that would also promote unity among American forces. The Committee on Public Information (CPI), under the leadership of newspaperman George Creel, had already established guidelines for American unity based on his interpretation of propaganda, which he defined as the “propagation of faith” in the cause [17]. In keeping with this purpose, Pershing endorsed the *Stars and Stripes* in its initial run on February 8, 1918, as a paper “written by the men in the service, [and one that] should speak the thoughts of the new American Army and the American people from whom the Army has been drawn” [18].

The *Stars and Stripes* was certainly not a trench journal, nor was it truly a unit magazine—unless one counts the entire AEF as a “unit”—but it contained the essential elements of both, along with a healthy dose of American propaganda. Produced by and for soldiers with the intention to improve morale, it maintained a continuous effort to shore up support for the officially sanctioned American cause. Beyond Pershing’s endorsement, the first issue sought to inspire confidence in the troops by pointing out the excellent medical care they could expect, noting that “if the incomes of all the well-known American specialists who have come to France to look after the health of the A.E.F. troops were lumped together, they would be enough to pay off the national debt [and] leave sufficient to satisfy a camp store-keeper” [19].

The editors’ choice to use a monetary measurement of medical skill is interesting and perhaps reflects a uniquely American character in 1917. If the wages of American medicos were not enough to impress, the paper assured its readers that the hospital system prepared by the AEF was more than up to the task by pointing to American ingenuity and industry. From the new folding litters that could go around corners in the trenches to the network of dressing stations, motor ambulances, evacuation hospitals, and surgical centers at base hospitals staffed by some of the best medical minds America had to offer, the Medical Department was prepared for the war, or so the *Stars and Stripes* argued.

This piece and others like it were classic sales pitches, pushing propaganda to inspire confidence. And unlike their British and French allies—who had grown disenchanted with their respective governments’ propaganda after more than a year of trench warfare—the Americans had not yet grown disillusioned with the official line.

Indeed, many of America’s best medical minds were certainly involved in the war effort, some well before the United States entered the war. George Crile of Cleveland’s Lakeside clinic and Harvard’s Harvey Cushing had been working with their French and British counterparts since 1915 to develop new surgical techniques at frontline hospitals. The Rockefeller Institute for Medical Research provided material support to French physician and Nobel Prize winner Alexis Carrel, who worked at the Rockefeller Foundation before the war broke out, in order to run a hospital near the front lines. They also sent Henry Dakin, a biochemist working with the institute, who had perfected a solution of sodium hypochlorite, which killed bacteria without doing too much damage to healthy flesh [20]. The Carrel-Dakin solution—essentially a dilution of bleach—proved to be a revolution in aseptic wound treatment and was quickly adopted by physicians across Europe. It must have been excruciating for patients, but it prevented the infections that killed so many in previous wars.

The Red Cross began efforts to organize, fund, and supply base hospital units in association with the nation’s leading hospitals and medical schools in 1917. Base Hospital No. 1, organized out of New York’s Bellevue Hospital and located in Brest—often the first sight of French soil for arriving American troops—was the first to be established in November 1917, but much of the base hospital network was still struggling to get ready by May 1918. The case of Base Hospital No. 30 illustrates this point.

Organized under the auspices of the American Red Cross Society in the University of California’s School of Medicine in the spring and early summer of 1917, Base Hospital No. 30 did not receive orders to officially muster until November, with the delay due to bureaucratic congestion in the Army [21]. Consisting of 25 officers, 65 nurses, and 150 enlisted men, the unit finally received orders for France in April 1918 after 5 months of drill and training at Fort Mason in San Francisco. During a short holdover in New York City, the officers were able to attend clinics on the latest medical procedures, including the Carrel-Dakin course on aseptic surgical techniques and wound care and instruction on the treatment of pneumonia and meningitis at the Rockefeller Institute, before setting sail for France. They arrived at their duty station in the resort town of Royat on May 7, where they found the Army had secured a dozen hotels for the purpose of establishing a hospital. Lieutenant Colonel Eugene S. Kilgore, Medical Corps, felt compelled to report on the poor conditions of the hotels, noting particularly that the kitchens—often located in the hotel basements—were veritable dungeons in sore need of updating before the hospital could expect to feed patients [22]. Unfortunately, the state of the kitchens proved to be the least of the worries for the personnel of Base Hospital No. 30.

Within a few days of occupying the hotels secured by Army procurement in Royat, it became apparent that the water supply was poor, the electrical supply was worse, and even the hospital’s skeleton-crew operations were too much for the old hotels’ drainage systems, many of which relied on cesspools that often overflowed into the hotel basements, causing enormous problems in the kitchens. The unit’s staff had precious little time to overcome these complications as the first trainload of 360 patients arrived on June 12, well before the improvements were complete. Thankfully, most of the patients in the initial train were convalescent, but 5 days later, a second train arrived with wounded troops who were fresh from the fighting at Belleau Wood, including many surgical cases and victims of mustard gas exposure.

Despite their difficulties, the members of Base Hospital No. 30 managed to treat the wounded in a manner that lived up to the *Stars and Stripes*’s boast of exceptional American medicine. Between receiving their first patients in June 1918 and the hospital’s closure in January 1919, the staff improved the facilities, utilized the latest aseptic surgical techniques, contained the spread of acute infections throughout the Spanish Flu epidemic, and managed to put many men on the path to recuperation with few casualties. In his report on closing the hospital, Colonel L. D. Carter, Medical Corps, noted the high morale of the hospital’s remaining wounded:

Men who were…maimed for life were happy and cheerful and felt they had done their work well. Intense suffering was borne with patience and fortitude, and it was rare indeed that any of the petty complaints so common with the sick soldier were made. They were appreciative of all that was done for them, and they themselves were always ready to help or cheer a more unfortunate comrade. [23]

Whether or not the wounded soldiers actually bore their suffering with the patience and fortitude that Carter describes is difficult to confirm, but we can be sure that Carter’s description is not the whole picture. Carter was a career Medical Corps officer who had taken command of Base Hospital No. 30 only a few weeks prior to its closure. His job was primarily bureaucratic. He was to oversee the closure of the hospital and write an official report to his superiors for the record. The hospital had performed exceptionally and, therefore, received a glowing review. The state of the patients, however, depended on one’s faith in the cause of the larger Medical Corps mission, and this is reflected in the use of solemn, courageous, and paternalistic tones when referring to the wounded in Medical Corps reports. Carter’s comments are emblematic of the archetype of men who felt their sacrifice contributed to the greater good, who appreciated everything that was being done for them, and who cheerfully bore the burdens of their wounds and therapy. That may have been the case in some cases, but it fails to capture the whole story.

## WRITING FOR WOUNDED WARRIORS: BUILDING COMMUNITY IN RECONSTRUCTION HOSPITALS

With the closing of the base hospitals in France, America’s wounded joined the river of men making the westward journey across the Atlantic to return home. The troop ships that had borne them to Europe, like the USS *George Washington*, now carried them home again. It was a joyous time. The AEF was returning home victorious, and many wanted to tell their stories. Before the armistice, the *George Washington*’s crew was prohibited from mentioning in print the units they transported, their destinations, or even the name of their own ship for fear of giving information to the enemy. Now, with the lifting of censorship restrictions, the crew could fire up the ship’s printing press and produce a newspaper with a new daily edition to entertain their passengers and provide the soldiers and sailors with an outlet to boast about their accomplishments.

*The Hatchet* was a small but professionally done newspaper, primarily consisting of two pages, each with a three-column spread. Front-page columns relayed the news coming over the ship’s wireless telegraph alongside stories about the units and interesting passengers aboard the ship. On the reverse, each edition had an editorial, a column of official announcements from the ship’s captain, advertisements for entertainment, sports results, and readers’ contributions from the passengers and crew. Columns of the latter variety provided brief glimpses into the mindset of the veterans who were returning home. Most were congratulatory in nature, boasting the accomplishments of the AEF in defeating the Kaiser’s well-steeled legions.

Occasionally, stories of a more somber variety appeared, as was the case in the edition of December 19, 1918. The paper ran a piece titled “World Series,” in which the author creatively described the war as if it were a baseball game in which the German team had just struck out the Allies’ Russian batter when “Sammy,” the Allies’ pinch hitter, stepped up to the plate:

His eyes are keen, his spikes are sharp, he’s filled with the courage of youth. Democracy gleams in his clear gray eyes, and his bat bears the trademark of truth. Now this is as far as the game has advance[d], so of course we can tell [no] more. But soon every fan in this troubled land will know the completed score. [24]

The editors noted that the author might have been traveling home aboard the *George Washington* but for the fact that he made his final sacrifice several months before the “game” was finished. Publishing the incomplete composition—no doubt a contribution from the anonymous author’s friends—was *The Hatchet*’s way of honoring the fallen and calling for a moment of reflection among the passengers. Many of the soldiers returning to the United States were indeed thrilled at the war’s end and proud of their accomplishments, but many were also likely mourning the loss of friends in France. Articles like “World Series” allowed them to communally address such grief while simultaneously recognizing their accomplishments. The passengers of the *George Washington* knew the final score, and they knew the cost.

Between December 1918 and November 1919, the *George Washington* made 20 transatlantic voyages, transporting 85,140 passengers—48,772 on the homeward voyages, including 4,680 wounded soldiers [25]. During the war, it braved stormy seas, submarine attacks, and the influenza pandemic of 1918, which claimed 89 lives among the passengers and crew. After the armistice, the *George Washington* and her crew celebrated 10 homeward journeys on the pages of *The Hatchet*, which was the last military newspaper that most of her passengers read before returning to civilian life. But for the wounded and the members of the Medical Corps who cared for them, the voyage home was just the next step in a war that would continue.

For many wounded troops, the experience of coming home again must have seemed a strange and lonely process. Since their military service began, these soldiers, sailors, and marines had been with their units, which became a surrogate family during training, deployment, and the war itself. Once wounded, however, their ties were effectively severed from the military family that sustained them to that point. They were evacuated from the battlefield to a collection station, then an evacuation hospital, and then a base hospital, where they would be stabilized for the next steps. Those unable to return to duty due to their injuries faced further evacuations via hospital trains and aboard ships like the *George Washington*, with every step in the process representing another separation from another temporary family formed by their fellow patients and caretakers. Once back in the United States, they were sorted and sent to a general hospital, often according to their wounds, which became intrinsic parts of their new identity.

US Army General Hospital No. 3—located in Colonia, New Jersey, about twenty-two miles from New York City—specialized in surgical cases for injuries to the extremities. It featured eleven acute surgical wards, including a neurological group; an X-ray unit; and its own artificial limb service and workshop [26]. Many men with previous amputations, gunshot or shrapnel wounds requiring reconstructive surgery, or head trauma found themselves transferred to this facility after it went operational in May 1918.

Patients soon discovered that the Reconstruction hospital, like the trenches, was a place of manifold stress and boredom. Rather than worrying about artillery, gas, and machine gun fire, they worried about surgery, infection, and pain. And they spent a great deal of time in bed with nothing to do. So when a group of convalescent patients and the post chaplain proposed the publication of a newspaper to improve morale with encouragement from the surgeon general’s office, the post commander was quick to approve the request, provided that the paper could pay its own way. With the support of local businesses that chose to advertise in the paper, *Over Here: The Official Publication of U.S. Army General Hospital No. 3* went to press on November 28, 1918, just as waves of wounded soldiers began crossing the Atlantic.

The editorial staff ensured its readers that “‘OVER HERE’ hopes to offer a word of cheer whenever possible and reflect truly the spirit of General Hospital No. 3. It plans to give record to the small, yet important happenings in the lives of those about us, [and] it longs to be regarded as Your paper” [27]. They understood that the men convalescing in the hospital’s wards were likely to despair of their situation, and they effectively appealed to the patients’ masculinity, humor, and sense of community with their fellows to alleviate this distress. In an article titled “Laugh and Live,” the paper’s editors sought to establish the parameters of the community forming at General Hospital No. 3:

There are very good reasons for believing that the ordinary civilian fosters the idea that well-fitted Army Hospital is a place of mournful meditations…We healthy ones, who have been privileged to operate here without being operated upon, hold far different views—and so do the greater portion of the patients. They themselves would be the last to admit the ownership of a gloomy thought…They would tell you, “It might be worse,” no matter what your question was and they would probably offer you a bit of philosophy to the effect that because a man loses a leg, it does not follow that he has lost a taste for dancing. [28]

The editors appealed to the wounded soldiers’ identity as soldiers—something special and set apart from the average citizenry through their shared military experience—and then built upon this communal identity by suggesting that members of this community should not entertain despair, for who better than soldiers who had seen combat understood that their situation could be worse? Who better than veterans understood that disability was merely another obstacle to be overcome? This reasoning certainly reflected Medical Corps propaganda, but it also appealed to soldiers’ senses of individual pride and communal responsibility to effectively boost morale. And lest the soldier-patients should feel they were merely being sold a bill of goods by some faceless official, the paper’s editors offered a clarification in the same article:

Perhaps a visitor would consider our staff an odd assortment of humans. There is an associate editor, for instance, who recently returned to An Atlantic Port after spending an interesting summer in France. [One] day, while strolling about the country near Chateau-Thierry, he picked up a piece of shrapnel with his right hand. It took two surgeons and five weeks to make him let go of it—now he writes with his left hand. Another of our stars left a considerable portion of his feet outside the trench one night; now when he wants the ’phone or the paste he simply reaches it with his crutch instead of walking around the table.

Many of the hospital’s patients would quickly spot the tongue-in-cheek humor of the editors’ supposed good spirits mixed with a genuinely positive, yet pragmatic outlook. They would also note that at least some of the men working on the paper were also recuperating from injuries and adjusting to serious disabilities. Thus, *Over Here*’s editors established rapport with their audience and were able to provide all the things in the hospital setting that made trench journals so effective on the front lines: a source of entertainment to pass the time, information to alleviate fear of the unknown, and a medium of individual expression and community building.

Like other medical unit newspapers, *Over Here* provided helpful information about the efforts to assist disabled soldiers’ in their return to civilian life in accordance with the directives of the War Risk Insurance Act (WRIA) of 1917. In every edition of the paper, readers found articles about vocational training opportunities at the hospital or in the surrounding area that would, in theory, help them find employment once they finished their rehabilitation to the Medical Corps doctors’ satisfaction and received their discharges. They could also read about the latest medical practices being employed to help them in their physical recuperation such as advances in anesthesia, surgical methods, and pain management, which was not only intended to be informative, but also to alleviate patients’ anxieties about their recovery. But perhaps the most effective support was found in the contributions of other patients.

*Over Here* and publications like it in general hospitals across the nation encouraged patients to contribute content for the publication: to take part in the communal construction of the paper and in defining the values of those in the hospital. Poetry was a common submission in every trench journal and unit magazine during the war. In the following piece titled “The Soldier,” the authors spoke of the meaning of sacrifice and reflected a sentiment shared by many wounded veterans.

“The Soldier”
The bars upon your shoulders, or the uniform you wear,
Doesn’t mean that you’re a soldier in this wide world war affair.
For the man’s a man in battle and your uniform so bright
Isn’t worth an empty cartridge if you don’t stand up and fight.
The title “soldier” should be sacred and not called to every one
Who sports a classy uniform, or totes around a gun.
Once I saw a soldier dying; (Yes, he’s worthy of that name)
Just an ordinary private, but, by God, he sure was game.
And before the last call summoned him to pass his last review,
He shook me by the hand and said, “Good-bye, old pal, to you.
“Tell my sweetheart that I loved her, God bless my little Jane;
Tell my mother I died smiling and I did not feel the pain.”
Gee, I envied him his rating, for he died and didn’t flinch,
Though his heart inside was bleeding—that’s a soldier, every inch.
And I know another soldier, though she never fired a gun,
And she never saw the trenches, and she never killed a Hun,
She’s the mother of the soldier I saw dying over there;
She’s a sort of super-soldier for she more than gave her share.
She gave her country all she had—her pride, her love, her joy;
She’s the highest type of soldier for she gave her only boy. [
29]

Sentiments such as those expressed in this poem likely resonated with the soldier-patients of General Hospital No. 3 on several levels. Many of the men would have lost friends in the fighting that shattered their own bodies. Poetry like “The Soldier” encouraged these men to frame such deaths in honorable trappings. It also appealed to their sense of masculinity. It reminded them of their duty to protect women and honor the family unit with the references to Jane and the sacrifices of the soldier’s mother. Further, it challenged them to look beyond their own complaints. The soldier did not succumb to pain. He could not avoid dying, just as many of the readers could not avoid their disabilities, but he died on his own terms, showing strength. This was an invitation for soldiers to look beyond their present complaints and say, “It might be worse.” Reader contributions like this are common in the pages of trench journals and hospital newspapers alike, though not all are so somber.

Humor played an invaluable role in maintaining morale, building community, and providing entertainment. It also played an important role in the healing process and was, therefore, highly regarded by everyone from the ward nurse to the surgeon general in the hospital system. Patients often passed the time by pulling pranks and playing games, some of which found their way into the paper’s pages. An article on January 10, 1919, provided information on a new disease that curiously only presented symptoms in the presence of visitors—often resulting in the visitor being subjected to ever more extraordinary tales of how the patients came to be in the hospital.

Private Wilson, who was no nearer the front than the quartermaster’s depot on the French coast, will pull from under his pillow the notched stick that is the particular delight of his ward. The bigger he makes the yarns that follow, the more entertaining to himself and all his wounded fellows within hearing distance. “Yep,” Private Wilson will begin, “ever’ last one of them notches means a dead German. Good thing the armistice was signed or I’d have to get me a pole.” [30]

The editors dubbed the disease “Kiddemallitis,” and it was “often found in the most virulent stages on the sidewalk in front of the hospital,” where convalescent soldiers gathered to meet the shop girls at noon. For example, “One chap with a leg gone tells how his late uncle bequeathed him $150,000 on condition that he be married by his twenty-fifth birthday,” and there he was, 10 days away from the date and wounded so he cannot go out to find a bride. Hospital officials generally allowed the men to play their games, but an officer noted that “we try to stop yarns like the one about the $150,000 going to blazes because of no girl. The fellow that started telling it was a handsome brute anyway. With his absent leg to add sympathy…it simply wasn’t right to make one girl endure it, and he was shooting it out to crowds of ’em.” Humor like this could disarm tense situations, humanize Reconstruction hospital experiences, and build a sense of community in addition to providing entertainment—all of which became ever more critical as the war’s Reconstruction phase continued through the summer of 1919.

While practices at Reconstruction hospitals varied, it is important to remember that these remained military institutions, subject to military discipline. If the advertisements in *Over Here* provide any guidance, convalescent patients and Medical Corps personnel were relatively free to come and go as they pleased between the hospital and the nearby town of Rahway, New Jersey, provided they performed their assigned duties, informed supervisors, and otherwise obeyed the rules. Trips to town allowed military personnel to get a taste of civilian life, such as shopping in well-stocked stores, dining at restaurants, and browsing the latest fashions. Yet, frequenting Rahway likely also reminded the men assigned to the general hospital—both patients and enlisted—that their war was not yet done, which was frustrating.

Occasionally, frustrations boiled over. On June 9, 1919, a number of enlisted men from the hospital managed to get in a fight at the Rahway carnival grounds, possibly with a group of local firemen [31]. In response, the hospital’s commanding officer restricted all enlisted personnel from traveling to Rahway except on official business, and the order was still in effect by the publication of the June 13 edition of *Over Here*, which printed a clarification of the order’s implications [32]. While the cause of the unrest among the enlisted at General Hospital No. 3 was not explicitly stated, the paper provided insight by collocating the Rahway restriction piece with a statement from the surgeon general:

We can now see the end of our work. All the battle casualties have been returned from France with the exception of 1,000…I realize that there is a great deal of dissatisfaction among the men…They naturally are anxious to get back to civilian life…There is, however, a situation to be faced which is simply this—the men who are coming back wounded, [who] sacrificed their body for civilization, must be cared for. This is the sacrifice on the part of the men in the Medical Corps. [33]

The meaning was clear: the war was not yet over for the Medical Corps, and while that was certainly frustrating, that was the job, and the job would be done. In keeping with military tradition, the entire body of enlisted personnel in a unit faced collective punishment for the actions of a few. This was easier on the commanding officers, but it certainly did not help alleviate issues of morale.

The enlisted personnel were not the only ones growing frustrated with continued military discipline. Many of the patients chaffed at their lack of freedom as well. Complaints about formations, inspections, ceremonies, meals, and restricted liberties were common in hospital newspapers, and the channels through which these grievances could be addressed were few. When the chain of command refused soldiers’ requests, the soldiers often turned to family contacts to seek help. One soldier wrote his mother, informing her that he had been transferred to the hospital near Rahway. He complained that the hospital was terrible: “Not a single place of amusement in sight and nothing but 2,500 one-legged men to look at. Nothing but woods, a wonderful place to keep us so that many Americans will never see the truth” [34]. This disgruntled soldier went on to complain that his request for a transfer to a hospital near his home had been denied, and though he now had an artificial leg, it was made up of three different types, which failed to hold up as he thought it should. And while he admitted that he was learning to walk again faster than expected, he hoped that his mother would write to the commanding officer and to their representatives to assist his situation. The soldier’s mother wrote the commander and included a copy of her son’s letter, which was how it came to appear in the newspaper, sanitized of the soldier’s name, though clearly the soldier and his commander would know its provenance.

Alongside the letter from the disgruntled soldier, the editors of *Over Here* published another letter, this one from the father of another soldier convalescing at the hospital. The father expressed gratitude for the fine treatment the hospital provided his son. No additional commentary accompanied the notes, and readers were left to decide for themselves what to make of these contrasting messages. That the negative letter was published at all, however, speaks to the candid transparency found in the smaller hospital publications. Such pessimistic missives were not published in the pages of the *Stars and Stripes* or *Carry On* unless they were subsequently characterized as the words of a coward, a malcontent, or as examples of what not to do.

Regardless of soldiers’ efforts to find relief by enlisting help from outside the military—from families, politicians, or groups of concerned citizens—such attempts were usually fruitless. Immediate relief from the Reconstruction hospital system was impossible as the laws were clear. No disabled soldier was to be discharged from the military while the possibility remained of improving their physical condition that would allow them to return to civil life as productive members of society. If the soldiers wanted to change their situations, they would have to find a way to reform the law. Ironically, what the veterans sought to change was itself a reformation of veterans’ entitlements.

## THE GREAT PENSION DEBATE: POLITICS, MEDICINE, AND AGENCY IN REFORMING VETERANS

Understanding the Reconstruction chapter of the First World War requires examining the historical precedents that shaped its sociocultural context. The Reconstruction hospital system was a novelty in the First World War, but one that emerged incrementally from the long history of veterans’ entitlement in the United States. In 1636, New Plymouth colony’s legislature passed an act providing support for soldiers who served and were subsequently disabled in the Pequot War [35]. This set a precedent that the Continental Congress built upon in their effort to encourage enlistments during the Revolutionary War. The Second Congress passed laws providing lifetime monetary compensation for officers [36], and years later, the Fifteenth Congress extended such pensions to all veterans of the conflict [37].

During the American Civil War, the government once again extended generous pensions to incentivize enlistment, and though those efforts failed to procure the numbers necessary to sustain the Union Army, the government nevertheless lived up to its promise, framing the pensions as “the patriotic duty of a grateful people to the relief of those mutilated in the preservation of the nation” [38]. But between 1865 and 1915, public attitudes toward the pension system shifted dramatically, resulting in strong calls for reform.

The Grand Army of the Republic (GAR)—an organization of veterans who served in the Union Army—became a powerful political lobby in the years between the Civil War and the Great War, and they found a receptive audience in the Republican Party. Republican politicians were more than willing to wave the bloody shirt to support expanding pensions to broader segments of the veteran population, a cause for which the GAR lobbied heavily. The 1862 General Law provided pensions only to veterans who sustained direct war-related disabilities. By 1873, Congress extended the definition of “war-related” to include conditions that presented themselves well after the war, such as arthritis or tuberculosis, and in 1879, they passed a bill providing pension payments in arrears. An entire industry of pension agents sprung up, full of men willing to assist veterans with pension claims in return for a cut of their proceeds, and the pension expansions continued. By 1915, all a soldier had to do to receive a lifetime pension was serve in the military for ninety days and live past the age of sixty-two or develop a disability of any sort in the interim [39].

The Progressive Era (1890–1920) was marked by efforts to address economic and social problems through the application of science and education. Muckraking journalists like Upton Sinclair, Lewis Hines, and Ida Tarbell led the way in exposing problems to public scrutiny and subsequent political reforms in food safety, child labor, and political corruption. Walter Hines Page led the charge against the veterans’ pension system. Page was a journalist who grew up in North Carolina after the Civil War, resenting the pension system because, in his mind, it continued and exacerbated the sectionalist wedge that divided the country. Moreover, he felt the pension system was not only fiscally irresponsible and rife with corruption, but that pensions undermined veterans’ potential contributions to society beyond their service.

Page and other anti-pension journalists relentlessly attacked corruption in the pension system, but such arguments found little traction in a nation going through a “masculinity crisis.” Several factors contributed to the perceived threat to American manhood: the enfranchisement of women, the “easy” life of middle-class work, immigration, and the closing of the frontier, to name a few. In response to this crisis, popular figures like Teddy Roosevelt glorified war, arguing that the rigors of battle developed bonds of fraternity, revealed a man’s mettle, and imparted the values of the “strenuous life” [40]. Framing masculinity this way made Civil War veterans into symbols of male virtue and patriotic vigor, making it difficult to attack corruption in the pension system. Ironically, it was precisely this masculine framing of veterans that provided anti-pension activists like Page with the type of rhetorical ammunition they needed to secure reform.

In a series of articles for Page’s *The World’s Work* magazine, famed *New York Times* journalist William Hale lambasted the pension system as a crime against the US Treasury and the memory of true Civil War heroes—the type of men “who had seen carnage and wrought it.” By 1910, Hale argued, the pensioner was more likely to be a man like Henry J. Hootman, who served with the 191st Ohio Volunteers for a period of eighty-three days in 1865—just seven days shy of qualifying for a pension under the Sherwood Act. Luckily for Mr. Hootman, a lame-duck Congress was more than willing to push through special pension petitions such as his. The problem, according to Hale, was that Hootman spent his last forty-two days of “service” laid up in the hospital with the measles before being mustered out.

Hale’s message was crystal clear: While deserving veterans were worthy of praise and compensation, after fifty years of pension expansions, the system was so rife with abuse that it was allowing the undeserving to collect lifetime pensions to live easy lives freed from their responsibilities [41]. Sham pensions like Hootman’s, thus, represented a disservice to both the taxpayer and the memory of the worthy veteran, while they simultaneously emasculated the corrupt pensioner, turning an otherwise able-bodied worker into a ward of the State. By 1916, the costs of the Civil War pension system had exceeded the cost of the war itself [42]. With the Great War looming and likely to involve millions of US soldiers, the government began exploring ways to reform veterans’ entitlements.

President Wilson turned to Judge Julian W. Mack and Children’s Bureau Director Julia Lathrop—both good progressives—to develop a new system of veterans’ benefits on the basis of worker’s compensation laws. The result was the WRIA of October 1917 [43]. The WRIA authorized funds to create a large-scale health care and vocation training system, and it compelled disabled soldiers to remain in military service while they underwent rehabilitation with the goal of returning “crippled” veterans to productive civil life [44]. Thus, the WRIA represented a major reframing of veterans’ entitlements along a paternalistic model that would, in theory, return veterans to economic independence and the virtues of work. It would also theoretically save the US Treasury a great deal of money in the bargain.

While the legislation’s stated aims were clear, the bill was vague in specifics regarding how those goals were to be achieved, so the Army Medical Corps turned to the nation’s top medical minds in emerging specialties. Surgeon General William C. Gorgas recruited specialists in psychiatry, neurology, psychology, ophthalmology, occupational and physical therapy, and orthopedics to oversee the details of creating the hospital system that would carry out the work [45]. By the end of 1917, the reformed policy was in place and medical professionals were helping the US Army develop the necessary infrastructure. What remained to be seen was how affected veterans would react to this reconceptualization of their entitlements. It is important to note that the WRIA did not change entitlements for veterans who were already receiving pensions; only those not yet in the pension system would be affected.

For the most part, veterans of the Great War appeared to accept the new benefit system. Medical Corps propaganda appealed to their sense of masculinity and individual responsibility to promote the new model. Veterans appreciated the government-provided vocational training and job placement—particularly the jobs in the government’s civil service, where the prospect of a generous pension remained. But they noted that the system was not perfect. In the months and years after the First World War, too many disabled veterans were being discharged without the reasonable prospect of a job, and the system seemed to completely neglect soldiers who were suffering from shell shock—post-traumatic stress—with many such veterans ending up in children’s hospitals and asylums for lack of facilities to support them. While Great War veterans generally accepted the WRIA reforms, they also sought ways to improve upon them.

One promising route to achieve reform was to join veterans’ organizations like the Disabled American Veterans or the American Legion, an institution formed in 1919 and one that has exercised a profound influence on veterans’ policy ever since. At least one article in *Over Here* encouraged servicemen to pledge their support to the American Legion, which they promised would be “a potent factor and of great value in all public work and policies [and whose] continued effort and organization [for veterans] will become more evident as the wounded grow older and men seek from their Government opportunities for betterment of all social conditions” [46].

Indeed, the emergence of a new wave of veterans’ groups like the American Legion challenged the political framework of the Reconstruction hospital system by providing veterans with a political voice in the process. The political pressure generated by the lobbying efforts of these groups led to the establishment of the Veterans Bureau and the expansion of veterans’ benefits in the early 1920s [47]. These reforms, based on the WRIA’s precedent of government-provided health care as a substitute for pensions, formed the familiar foundations of the modern Veterans Administration [48]. But while Great War veterans’ efforts to flex their political muscles were just forming in 1919, the men and women of the general hospital system remained busy in the work of reconstructing and rehabilitating the remaining wounded.

General Hospital No. 3 continued its efforts in recuperating wounded soldiers until October 1919. Official US Army Medical Department reports provided all the information that the Army felt was pertinent to the implementation, function, and results of the hospital in its seventeen-month run [49]. But as the pages of *Over Here* demonstrated, the effort to rehabilitate America’s wounded soldiers extended well beyond the official reports and tabulated numbers. A community developed among the hospital staff, patients, aid workers, and local businesses that supported the paper and catered to its readership. While many eagerly anticipated the order to close the hospital by October 15, 1919, because it meant a long-awaited discharge and return to civilian life for most, that same order also meant the dissolution of the community that had sustained them all through the Medical Corps’ longest, hardest battle of the war.

In the final edition of *Over Here*, the editorial staff marked the occasion with almost sorrowful nostalgia. The editors noted the “unusual and thrilling fun” of living among so many people “waiting to see the small humors of their lives appear in print.” They remarked on the gratifying nature of being able to serve and gain the friendship of the remarkable patients, and of the honor of sharing the patients’ thoughts and helping them achieve “what was best for them.” They exclaimed over the skill and dedication of the surgeons, nurses, and aides who “devoted their skill to the physical and mental Reconstruction of the wounded.” And they reflected on the mission of the paper itself:

What has the Press accomplished, locally, during its 45 weeks of existence? Well, the results are doubtful. Mostly, perhaps, there has been the feeling of satisfaction that the paper has built a place for itself in this odd community…In [the first volume, this paper remarked that] it plans to give record to the small, yet important happenings in the lives of those about us…That was an ambitious mission. If we have failed, our friends have forgiven us. We could ask no more. [50]

General Hospital No. 3 represented a microcosm of the wider Reconstruction hospital system established during the Great War. It was part of a network of temporary specialized hospitals that were intended to rehabilitate the war’s wounded and return them to civil life as quickly as possible, and the US Army kept meticulous records of the hospital’s costs, specifications, operations, and effectiveness. The land upon which it was built was leased to the secretary of war for $1 a year, and the cost of construction totaled $2.75 million. It was built on the standard base hospital model but enlarged to accommodate the considerable number of surgical cases anticipated by the surgeon general’s office. Rather than the standard 500-bed pattern, General Hospital No. 3 boasted 1,700 beds in 30 wards. The hospital cared for an average of 1,300 patients at a time with a peak patient load of 1,944 in December 1918. It included a dental clinic, laboratory, X-ray department, specialized surgical wards, 3 recreational facilities, and an orthopedic workshop that created and fitted 843 artificial legs, 75 artificial arms, and 2,745 splits and braces. Between May 1918 and October 1919, surgeons performed 2,051 operations and the hospital treated 5,153 patients, successfully completing 4,571 cases—meaning that the patients either returned to duty, transferred to other facilities, or were successfully discharged. Of the 582 unsuccessful cases, there were 15 desertions, 22 deaths, and 545 patients discharged for disability [51]. These numbers tell the statistical success story of the Reconstruction effort in keeping with the stated goals of the WRIA. But the whole story goes well beyond the numbers.

## CONCLUSION: THE TRANSFORMATION OF WOUNDED SOLDIERS TO VETERANS AND THE PATH FORWARD

When the Wilson administration set out to reform veterans’ entitlements through the WRIA in 1917, it essentially set out to reform veterans themselves—politically, physically, and socially. The government employed progressive-minded experts in forming legislation and policy reform that dramatically transformed veterans’ entitlements in the United States, replacing the old and corruptible pension system with medical care and oversight. They empowered medical specialists in the Medical Corps with the authority to oversee the policy’s implementation, and a little over two years after the WRIA went into effect, these efforts to reshape wounded veterans’ bodies—while far from perfect—had proved remarkably successful in returning disabled soldiers to productive lives.

But reforming the social perception of veterans from the image of the corrupted grifter of 1910 to that of the honest and productive man of 1919 required a simultaneous transformation from below. Appealing to contemporary perceptions of masculinity, official propaganda outlined the goal, but it could only go so far in making that goal a reality. Just as the chaos of combat shattered the glorified images of war that decorated recruitment posters, idealized depictions of cheerful soldiers eager to overcome a missing limb and learn a trade could fall apart for men confronted with the uncertainties of reality.

Yet, in both combat and convalescence, the communities that formed between the soldiers sustained the majority of them in their struggles to endure the unendurable, and these communities are well described in both the trench journals and hospital newspapers produced by and for their members. These publications reflected the concerns and values of a changing readership—technically the same group of men, but one that changed in subtle but important ways. Trench journals helped frontline soldiers endure the hardships and horrors of combat. Similarly, hospital newspapers boosted morale among the disabled by facilitating community development and reminding the men that, while their war continued, they were not alone. And veterans’ organizations like the Disabled American Veterans and American Legion represented a continuation of these communities and a vessel through which to advocate for improvements in the new system of health care benefits.

As the Medical Corps general hospitals completed their mission and cased their colors, many of their patients and personnel were finally able to join the rest of the nation in a new era of relative peace after the longest and hardest battle of the war. Veterans of that chapter would go on to cement the foundations of the modern American veterans’ health care system. And while modern debates leave the future of that system in doubt, if history is a guide for the future, emerging studies into the role of veterans’ voices may prove insightful in the next chapter of this continuing story.

## 

**Aaron Jackson**, aaron.jackson@ucsf.edu, Doctoral (PhD) Candidate, Department of Anthropology, History & Social Medicine, University of California, San Francisco, San Francisco, CA
